# Supplementary material for: 2LE-BO-DeepTrade: an integrated deep learning framework for stock price prediction
Source: PeerJ Comput Sci. 2025 Aug 8;11:e3107. doi: 10.7717/peerj-cs.3107 (PMC12453771; doi:10.7717/peerj-cs.3107)
Supplement: Supplemental Information 6 [file peerj-cs-11-3107-s006.pdf]

Reproducibility

Computing Infrastructure: The experiments were conducted on a system running Windows OS, with hardware configurations including an 12th Gen Intel(R) Core(TM) i9-12900K CPU, 32GB RAM, and an NVIDIA RTX A2000 GPU. We used Python 3.10 and TensorFlow 2.x for development and experiments.

Model Description: The model takes historical stock price data as input, performs denoising using 2LE-ICEEMDAN, trains a deep learning model to predict future prices, and outputs buy/sell signals based on a PLR trading strategy. Specifically, it optimizes hyperparameters to improve prediction accuracy and profitability.

Evaluation Method: The primary evaluation metrics used were statistical metrics (RMSE, MAE, MAPE, and R-squared) and profitability analysis via a PLR-based trading strategy.

Assessment Metrics (Justification): Statistical metrics and profitability analysis were chosen as evaluation metrics because they directly reflect the performance of the model and the convergence of the training process.

Limitations/Validity: We discussed the model's generalization capability across diverse stock datasets and its potential limitations. The model's effectiveness has been rigorously validated through extensive experimentation.

Code and Data Availability: The code used in this study is available at https://github.com/aksehird/2LE-BO-DeepTrade/blob/main/2LE-BO-DeepTrade.py."
